# The Association between Uric Acid and Metabolic Syndrome in a Middle-Aged and Elderly Taiwanese Population: A Community-Based Cross-Sectional Study

**DOI:** 10.3390/healthcare12010113

**Published:** 2024-01-03

**Authors:** Chun-Ru Lin, Po-An Tsai, Che Wang, Jau-Yuan Chen

**Affiliations:** 1 Department of Medical Education, Chang Gung Memorial Hospital, Linkou Branch, No. 5, Fuxing Street, Guishan District, Taoyuan City 333423, Taiwanwulongtsai@cgmh.org.tw (P.-A.T.); chewang@cgmh.org.tw (C.W.); 2 Department of Family Medicine, Chang-Gung Memorial Hospital, Linkou Branch, Taoyuan City 333423, Taiwan; 3 College of Medicine, Chang-Gung University, Taoyuan City 333323, Taiwan

**Keywords:** uric acid, metabolic syndrome, cardiovascular risk factors

## Abstract

Background: Metabolic syndrome (MetS) is a prevalent health condition in Taiwan that places individuals at higher risk of cardiovascular disease, diabetes, and stroke. Therefore, the identification of risk factors associated with MetS is crucial. The aim of this study was to investigate the association of uric acid and MetS in a Taiwanese community with a middle-aged and elderly population. Methods: This cross-sectional study enrolled residents aged 50–90 years living in one community. All of the subjects received a standardized personal interview, including a structured questionnaire, anthropometric measurements, and blood samples were collected for laboratory testing. MetS was defined as excess waist circumference, high blood pressure, high blood sugar, high serum triglycerides, and low serum high-density lipoprotein (HDL). Multiple logistic regression models were used to evaluate uric acid tertiles associated with MetS. Results: A total of 400 subjects were enrolled in the analysis. The overall prevalence of MetS was 35.8%. The prevalence of MetS increased gradually with increasing serum uric acid levels (*p* value < 0.001). A significant association between uric acid and cardiometabolic risk factors was confirmed, with a Pearson’s correlation coefficient for waist circumference of 0.30 (*p* < 0.001), a coefficient for systolic blood pressure of 0.13 (*p* = 0.01), a coefficient for triglycerides of 0.33 (*p* < 0.001), and a coefficient for high-density lipoprotein of −0.30 (*p* < 0.001). The adjusted odds ratio (OR) of the high uric acid tertile level for MetS was 2.48 (95% CI = 1.31–4.71, *p* = 0.01). The area under the ROC curve (AUC) for uric acid in predicting MetS was 0.621 (*p* < 0.001). Conclusions: The prevalence of MetS in our study population is high. High serum uric acid levels are independently associated with the presence of MetS among the middle-aged and elderly Taiwanese population.

## 1. Introduction

Metabolic syndrome (MetS) is a cluster of metabolic dysregulation disorders, including insulin resistance, atherogenic dyslipidemia, hypertension, and obesity [1]. Although the complexity and multifaceted origin of MetS are not fully understood, it has been strongly suggested that sedentarism and unbalanced dietary patterns may play an important role in its etiology [2]. Individuals diagnosed with MetS were also found to exhibit a greater than twofold increase in risk for cardiovascular diseases [3] and cancers [4]. According to a partially nationally representative survey in China in 2009, the prevalence of MetS was 21.3%, and it was higher among people living in urban areas than in rural areas [5]. In addition, the prevalence progressed with age and was more prevalent in females than males in another nationally representative cross-sectional study in China [6]. Moreover, the prevalence of MetS has rapidly increased recently among people in the Asia-Pacific region, including in Taiwan, with a prevalence of 13.6% to 25.5% from 1993–1996 to 2005–2008, respectively [7]. In modern society, people tend to exhibit a sedentary lifestyle, which is a possible risk factor attributed to MetS [8]. Therefore, the enhancement of our knowledge surrounding MetS has become an important issue.

Hyperuricemia, which refers to increasing serum uric acid levels, has been frequently associated with MetS [9]. However, the definite relationship between uric acid and MetS is still unclear. In the past few years, hyperuricemia was attributed to the effects of insulin resistance, which reduced the urinary excretion of uric acid [10]. However, recent experimental studies have demonstrated that hyperuricemia may have a causal role in MetS [11]. In addition, uric acid can be considered an indicator of oxidative level, and an increase in uric acid concentration was shown to represent a status of active inflammation [12]. In a previous study, higher serum uric acid levels may have resulted in an increased risk of MetS; however, the correlation was not further examined [13].

Currently, the exact relationship between uric acid and MetS remains uncertain and controversial. Therefore, this cross-sectional study aimed to explore the association between uric acid levels and MetS in Taiwan. To allow for appropriate adjustment, a comprehensive sample of participants was included, and several relative risk factors were collected. In addition, we constructed receiver operating characteristic curves to assess the predictive ability of uric acid levels for identifying MetS in the middle-aged and elderly population of Taiwan. Our study aimed to identify and characterize potential associations between uric acid levels and MetS in Taiwan’s middle-aged and elderly populations.

## 2. Materials and Methods

### 2.1. Study Design and Participants

This study used a cross-sectional and community-based design. Participants were recruited from a health survey project that was completed in 2014 in the northern region of Taiwan. Our sampling framework was meticulously established to represent middle-aged and elderly individuals in Guishan district, Taoyuan City. We conducted a cluster-randomized selection across 28 villages. To be included in the study, residents had to be aged 50 years or older and have lived in the community for at least six months. Initially, a larger pool of residents was considered. However, we first excluded individuals who we were unable to contact, as well as those with disabilities and empty homes with no residents, which resulted in 619 ambulatory subjects who were eligible for participation. From these individuals, we further excluded individuals who declined to participate or who had a recent history of cardiovascular disease. After these exclusions, we successfully interviewed 400 subjects for our study. The sample size was determined using G*power software version 3.1.9.7 to achieve adequate statistical power. A calculated sample size of 352 was projected to provide 90% power to detect a twofold increase in odds ratio, with a baseline prevalence of the condition at 15% and an alpha of 0.05 for a two-tailed test, while also accounting for confounding factors with an R^2^ of 0.5. With a final sample size of 400 participants, our study exceeds this threshold, thus highlighting that our research possesses sufficient statistical power for the conducted analysis. All of the participants carefully answered a detailed questionnaire containing their personal information and relevant medical history during the health survey. Blood and urine tests were performed to obtain essential biochemical data from each participant. Based on uric acid levels, the sample of 400 participants was divided into three different groups. Ethical approval for the study was obtained from the Linkou Chang Gung Memorial Hospital Institutional Review Board (102-2304B), and all participants had signed informed consent.

### 2.2. Data Collection and Measurements

Data collection included a substantial questionnaire designed to collect pertinent information such as age, sex, alcohol consumption patterns (i.e., frequency of drinking of more than two days per week), and current smoking behavior (i.e., active smoker or nonsmoker). Participants self-reported both alcohol consumption and smoking status. In addition, the sedentary time of the participants, which is referred to as sitting time, was determined by self-reporting. Furthermore, medical records were reviewed to obtain information on prevalent diseases such as MetS, hypertension (HTN), diabetes mellitus (DM), and dyslipidemia. Body mass index (BMI) was calculated by dividing weight in kilograms by the square of height in meters. The systolic blood pressure (SBP, measured in mmHg) and diastolic blood pressure (DBP, measured in mmHg) were measured several times after a resting period. Waist circumference was determined as the measurement taken midway between the iliac crest and the last rib while standing in a horizontal plane. Analyses of the participants’ biochemistry using the Roche cobas^®^ Connecting Module (CCM) were performed in the Roche model laboratory at the Taiwan E&Q Clinical Laboratory. Laboratory data included measurements of uric acid (mg/dL), triglycerides (TG, expressed in mg/dL), fasting plasma glucose (FPG, expressed in mg/dL), low-density lipoprotein (LDL-C, expressed in mg/dL), high-density lipoprotein (HDL-C, expressed in mg/dL), and alanine transaminase (ALT, expressed in mg/dL).

### 2.3. Definition of MetS and Other Variables

According to the Asian World Health Organization (WHO) criteria, MetS is indicated if three or more of the following five criteria are met: (1) waist circumference over 90 cm in men or 80 cm in women, (2) blood pressure over 130/85 mmHg or the use of drugs for HTN control, (3) TG level over 150 mg/dL, (4) HDL-C level less than 40 mg/dL in men or 50 mg/dL in women, and (5) FPG over 100 mg/dL or the use of oral hypoglycemic agents or insulin therapy [14]. DM was defined as having an FPG level equal to or greater than 126 mg/dL, a history of diabetes mellitus, ongoing insulin therapy, or the use of an oral hypoglycemic agent [15]. Dyslipidemia was operationally defined as an LDL-C level equal to or greater than 130 mg/dL, an HDL-C level less than 40 mg/dL in men or less than 50 mg/dL in women, a TG level equal to or greater than 150 mg/dL, a TC level equal to or greater than 200 mg/dL, or the use of lipid-lowering medication [16]. HTN was defined as SBP equal to or greater than 140 mm Hg, DBP equal to or greater than 90 mm Hg, or the use of antihypertensive medication to treat HTN [17]. We defined drinking status as alcohol drinking frequency equal to or greater than 3 days per week. We defined regular exercise as an exercise frequency equal to or greater than 2 days per week.

### 2.4. Statistical Analysis

The participants in this study were divided into three different groups based on their respective uric acid levels: low, middle, and high. Specifically, uric acid levels below 5.1, between 5.1 and 6.2, and above 6.2 were referred to as the low, middle, and high uric acid groups, respectively. Continuous variables, including age, SBP, DBP, BMI, WC, ALT, FPG, HDL-C, LDL-C, TC, TG, and uric acid, were reported as the means with their corresponding standard deviations (SDs). Categorical variables such as gender, alcohol consumption, current smoking status, regular exercise habits, MetS, HTN, DM, and hyperlipidemia were presented as frequency counts and percentages. Statistical significance was determined via one-way analysis of variance (ANOVA) for normally distributed data. Pearson’s correlation test was used to assess the association between uric acid levels and other variables, including age, SBP, DBP, BMI, WC, ALT, FPG, HDL-C, LDL-C, TC, and TG. Bar graphs were used to show the prevalence of MetS within each group. Logistic regression analysis was used to assess the association between MetS and uric acid levels by using MetS as the dependent factor. The Youden index and receiver operating characteristic (ROC) curves were used to determine the optimized uric acid cutoff value for identifying the risk of MetS. Statistical significance was set at a *p* value of less than 0.05. All of the statistical analyses were performed by using IBM SPSS Statistics version 20.0 for Windows (IBM Corp., Armonk, NY, USA).

## 3. Results

Table 1 reports the general characteristics of participants in the study. A total of 400 participants were included, including 130 individuals in the low uric acid group, 138 individuals in the middle uric acid group, and 132 individuals in the high uric acid group. The mean age of the participants was 64.47 ± 8.45 years. To compare the clinical and demographic data between the different groups, one-way ANOVA and chi-squared tests were used. Regarding the uric acid level groups, no significant differences were observed in age (*p* = 0.10), ALT (*p* = 0.12), FPG (*p* = 0.52), LDL-C (*p* = 0.33), TC (*p* = 0.50), regular exercise status (*p* = 0.63), or DM (*p* = 0.87). However, statistically significant differences were found for SBP (*p* = 0.04), DBP (*p* = 0.001), BMI (*p* < 0.001), WC (*p* < 0.001), HDL-C (*p* < 0.001), TG (*p* < 0.001), alcohol consumption status (*p* = 0.003), current smoking status (*p* = 0.003), MetS (*p* < 0.001), HTN (*p* = 0.01), and hyperlipidemia (*p* = 0.02). In particular, the high uric acid group had a higher frequency of chronic diseases, including MetS, HTN, and hyperlipidemia.

Table 2 reports the correlation between uric acid levels and multiple cardiometabolic risk factors. Uric acid levels were significantly positively correlated with SBP (r = 0.13, *p* = 0.01), DBP (r = 0.23, *p* < 0.001), BMI (r = 0.21, *p* < 0.001), WC (r = 0.30, r < 0.001), ALT (r = 0.1, 0.04), and TG (r = 0.33, *p* < 0.001), whereas uric acid levels were significantly negatively correlated with HDL-C (r = −0.30, *p* < 0.001). However, there were no significant correlations between uric acid and age, FPG, LDL-C, or TC.

Table 3 reports the odds ratios (ORs) of uric acid levels in association with MetS (both before and after adjusting for other potential risk factors) by using a multiple logistic regression model. The adjustment variables included age, sex, alcohol drinking, smoking, BMI, regular exercise, ALT, and TC. Before adjustments, there were significant differences in the middle uric acid group (OR: 2.02, 95% confidence interval [CI]: 1.18–3.44, *p* = 0.01) and the high uric acid group (OR: 2.86, 95% CI: 1.68–4.88, *p* < 0.001). After adjustments for these factors, there were no significant differences in the middle uric acid group (OR: 1.73, 95% CI: 0.95–3.16, *p* = 0.07), while there were significant differences in the high uric acid group (OR: 2.48, 95% CI: 1.31–4.71, *p* = 0.01). All of the odds ratios and corresponding *p* values decreased after adjustments for covariates in the logistic regression model.

**Figure 1:**
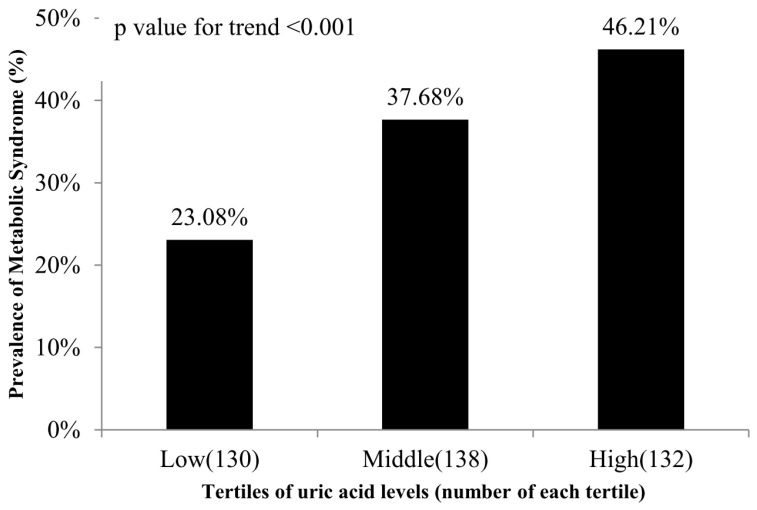
The prevalence of MetS at different uric acid levels.

**Figure 2:**
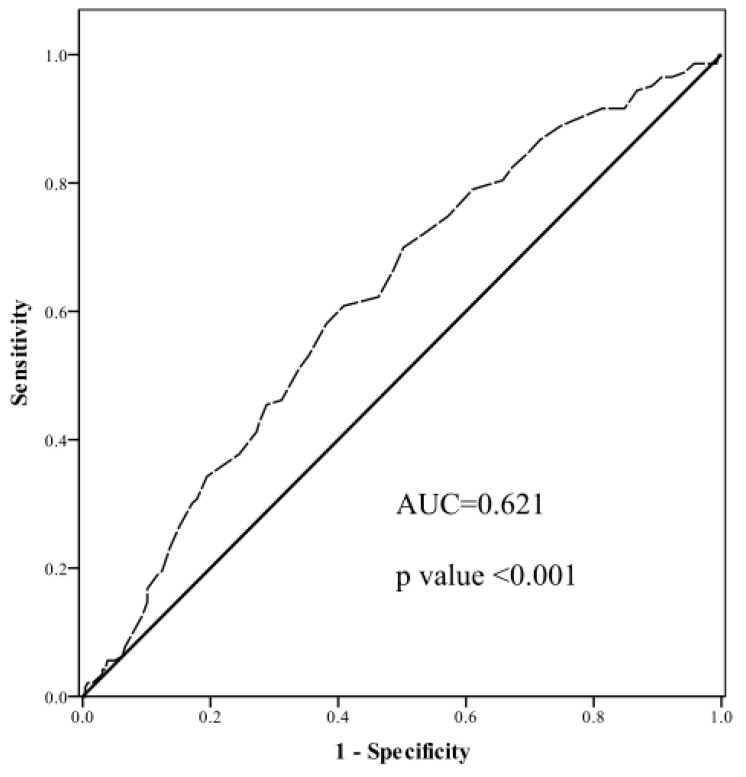
The area under the ROC curves of uric acid and MetS. The solid line represents the ROC curve for a random guess. The dotted line represents an example of a real method of classification.

Figure 1 reports the prevalence of MetS in different uric acid level groups. The high uric acid group had a prevalence of MetS of 46.21%, the middle uric acid group had a prevalence of 37.68%, and the low uric acid group had a prevalence of 23.08%. This result indicated a significantly higher prevalence of MetS in the high uric acid group than in the low uric acid group (*p* < 0.001). Figure 2 reports the ROC curve for the performance of uric acid levels as a biomarker for the identification of MetS. Table 4 reports the area under the ROC curve (AUC), sensitivity, and specificity using the optimized cutoff point for uric acid in predicting MetS. The AUC was calculated to be 0.621 with a cut-off point of 5.62, thus indicating moderate discriminatory ability of uric acid levels in the prediction of MetS (*p* < 0.001). In summary, our study showed significant positive correlations between uric acid levels and SBP (r = 0.13, *p* = 0.01), DBP (r = 0.23, *p* < 0.001), BMI (r = 0.21, *p* < 0.001), WC (r = 0.30, r < 0.001), ALT (r = 0.1, 0.04), and TG (r = 0.33, *p* < 0.001). Moreover, there was a significant negative correlation between uric acid levels and HDL-C (r = −0.30, *p* < 0.001). Furthermore, after adjustments for potential risk factors, both the middle uric acid group (OR: 1.73, 95% CI: 0.95–3.16, *p* = 0.007) and the high uric acid group (OR: 2.48, 95% CI: 1.31–4.71, *p* = 0.01) had a higher risk of MetS than the low uric acid group. In addition, the AUC for the association between uric acid level and MetS was 0.621 (*p* < 0.001).

## 4. Discussion

Our study showed significant positive correlations between uric acid levels and SBP (r = 0.13, *p* = 0.01), DBP (r = 0.23, *p* < 0.001), BMI (r = 0.21, *p* < 0.001), WC (r = 0.30, *p* < 0.001), ALT (r = 0.1, 0.04) and TG (r = 0.33, *p* < 0.001). On the other hand, there was a significant negative correlation between the uric acid level and HDL-C (r = −0.30, *p* < 0.001). Furthermore, after adjustment for potential risk factors, the high uric acid group (OR 2.48, 95% CI 1.31–4.71, *p* = 0.01) had a higher risk of MetS than the low uric acid group. In addition, the AUC for the association between uric acid level and MetS was 0.621 (*p* < 0.001) with the cut-off point of 5.62.

MetS is associated with both all-cause mortality and cardiovascular disease mortality, with hazard ratios of 1.08 and 1.35, respectively [18]. In 2018, the prevalence of hypertension was reported to be 24.1% [19]. Earlier research conducted in Taiwan in 1976 and 1984 reported the prevalence of hypertension as 14.1% and 14.8%, respectively [20]. The incremental change within that decade may not appear striking; however, it is the long-term shift that we aim to emphasize. The significant increase in prevalence from the late 20th to the early 21st century underscores a critical public health trend, which reflects the implications of demographic aging and evolving health risk factors over time.

In our research, we identified a linear correlation between uric acid levels and both systolic (SBP) and diastolic blood pressure (DBP). It is imperative to acknowledge the findings from a separate retrospective observational study in China that was conducted on 3345 males and 3774 females, which reported a positive association between uric acid and SBP in both males and females. For males, the association exhibited a β coefficient of 0.047 (*p* = 0.013), whereas for females, it demonstrated a β coefficient of 0.040 (*p* = 0.028) [21]. Furthermore, another retrospective cohort investigation conducted in China with 1082 participants observed a comparable positive relationship in the 41–50 age group concerning both SBP and DBP with respect to uric acid levels. Specifically, the β coefficient for SBP was 0.35 (*p* < 0.001), whereas for DBP, it was 0.29 (*p* < 0.001) [22]. Another noteworthy retrospective study conducted in Jiangsu Province, China, with 39,736 participants demonstrated a positive linear-like relationship between body mass index (BMI) and uric acid level. In comparison to individuals classified as being underweight (*p* < 18.5), those individuals falling within the normal range (18.5–23.0) exhibited an odds ratio (OR) of 1.558. In addition, individuals classified as being overweight (23–27.5) demonstrated an OR of 2.98, whereas those classified as being obese (≥27.5) exhibited an OR of 5.968 [23]. These findings suggested a progressive increase in the odds of being in a higher weight category as BMI increases.

Obesity and morbid obesity also showed an increasing prevalence in Taiwan over the periods of 1993–1996, 2005–2008, and 2013–2014. The prevalence of morbid obesity was found to be 0.4%, 0.6%, and 1.4%, whereas the prevalence of obesity was reported to be 11.8%, 17.9%, and 22.0%, respectively [24].

A study conducted in Taiwan between 1993–1996 and 2005–2008 indicated distinct trends in the prevalence of hyperlipidemia and hypertriglyceridemia. Although there was an overall increase in these conditions, the patterns differed between genders. For hyperlipidemia, the prevalence among males increased from 10.2% in the first period to 12.5% in the second. In contrast, females showed a slight decrease from 11.2% to 10.0% over the same time period, thus suggesting varying impact factors or interventions between the genders. In the case of hypertriglyceridemia, both males and females exhibited an increase in prevalence, with males exhibiting an increase from 13.4% to 20.8% and females exhibiting an increase from 6.1% to 7.9%. These findings highlight the complex and gender-specific nature of metabolic disease trends in Taiwan [25]. In a longitudinal population-based epidemiological study conducted in China with a sample size of 4190 subjects, there was a significant relationship between elevated uric acid levels and increased incidence of hypertriglyceridemia. The cumulative incidence of hypertriglyceridemia was found to be 28.2%, 29.1%, 36.9%, and 45.6% across quartiles 1, 2, 3, and 4, respectively (*p* for trend < 0.001) [26].

The prevalence of DM in Taiwan increased from 5.8% to 9.4% over the time period of 2005–2014 [27]. It is imperative to enhance awareness and prioritize attention toward the escalating prevalence of metabolic diseases in Taiwan. Our research provides one aspect of the entire view and provides access to resolving this problem.

In an earlier case–control study conducted in Iran involving 101 participants, the investigators aimed to explore the association between serum uric acid levels and components of MetS. The notable observations from their study align with previously mentioned findings. Their study demonstrated that as the number of metabolic factors increased, there was a corresponding increase in mean serum uric acid levels. Additionally, subjects with abnormal TG levels, increased waist circumference, low high-density lipoprotein cholesterol (HDL-C) levels, and elevated blood pressure exhibited higher serum uric acid levels than those with normal levels. Specifically, the mean increases in uric acid levels were 22.8, 21.4, 14.4, and 9.4 micromol/L, respectively (*p* ≤ 0.001) [28]. Our study found that people with higher uric acid levels have a higher risk of MetS. A previous meta-analysis of prospective studies conducted in 2015 found that individuals in the highest uric acid group had a combined relative risk (RR) of 1.72 compared to those in the lowest uric acid group [13], which is similar to our results. In addition, in our research, the AUC was calculated to be 0.621, thus indicating moderate discriminatory ability of uric acid levels in the prediction of MetS (*p* < 0.001). In a previous 10-year longitudinal study, a total of 8005 subjects were randomly selected. The study determined that the optimized cutoff point for males was 7.3 mg/dL, whereas for females, it was 6.2 mg/dL. They found that uric acid levels could predict MetS in men (hazard ratio: 1.658; *p* < 0.05) [29], which was similar to our findings.

The pathophysiological relationship between MetS and uric acid is an area of ongoing research, yet some progress has been made. An article summarized that excessive fat storage is a major contributing factor to MetS, and studies have demonstrated that uric acid plays a significant role in this mechanism. When animals accumulate excess fat, it is not only stored in adipose tissue but also in the liver and serum as triglycerides. This fat accumulation is often associated with insulin resistance and elevated blood pressure. The underlying mechanisms involve nucleic acid metabolism, whereby the stimulation of adenosine monophosphate (AMP) deaminase promotes fat storage and insulin resistance. Furthermore, activation of AMP-activated protein kinase facilitates fat breakdown and reduces the production of glucose. Uric acid, which is a product of AMP deaminase, has been identified as a key factor that promotes fat storage. It is implicated in the pathogenesis of MetS by contributing to insulin resistance and fat accumulation [30]. These findings suggest that the metabolic effects of uric acid may play a crucial role in the development and progression of MetS. Recent studies have illuminated the pathophysiological cascade originating from abdominal obesity that leads to elevated uric acid levels, which correspondingly heightens the risk of hypertension, diabetes mellitus, and ultimately, metabolic syndrome. Abdominal obesity is closely linked to increased uric acid levels due to its association with insulin resistance and adiposity [31,32]. This elevated uric acid level contributes to endothelial dysfunction and vascular remodeling, which are key factors in the development of hypertension and cardiovascular diseases [33,34,35]. Furthermore, the impact of uric acid on metabolic health is observed in its predictive capacity for metabolic complications, thus highlighting its role in the progression of diabetes and metabolic syndrome [36,37]. Therapeutic studies, such as those focusing on empagliflozin, have demonstrated how the management of uric acid levels can aid in controlling these metabolic risks [38]. Finally, the importance of monitoring uric acid from an early age is emphasized, as its elevation due to obesity can set the stage for future metabolic disorders [39].

The URRAH project, which is similar to our research, has extensively explored the relationship between serum uric acid (UA) and cardiovascular (CV) diseases. As highlighted in their recent review, URRAH identified UA as being an independent predictor for various CV conditions, including all-cause and cardiovascular mortality, acute coronary syndrome, stroke, and heart failure [40]. Although our study focused on the Taiwanese population, it mirrors these broader findings by examining the role of UA in metabolic syndrome and its associated complications. This parallel design allows our research to contribute valuable insights that are specific to our regional context while also aligning with the larger global understanding of the impact of UA on metabolic and cardiovascular health. The comparative analysis emphasizes the relevance of our findings within the wider spectrum of international research, thus showcasing the importance of studying these relationships across diverse populations.

This study had several limitations that should be acknowledged. First, the sample size was small, which may affect the statistical power and generalizability of the findings. In addition, the study focused exclusively on the Taiwanese population, which limits the ability to extrapolate results to other regions or ethnic groups. Ethnic differences may influence the relationship between the examined variables, and caution should be exercised when applying the findings to populations of different ethnicities. Furthermore, the study specifically included only elderly individuals, and the results may not be applicable to other age groups. Age-related physiological changes and health conditions in the elderly may differ from those in younger populations, thus necessitating separate investigations for different age cohorts. Moreover, the cross-sectional design of the study is a notable limitation. It precludes us from establishing any cause-and-effect relationships, as it only provides a snapshot of associations at a single point in time. The inferences drawn from such designs regarding causality must be considered with considerable caution. To truly understand the dynamics between uric acid levels and metabolic syndrome, longitudinal studies are indispensable. These studies would permit the observation of changes over time, thus providing insights into causality that our cross-sectional study could not. Future research should not only be longitudinal in nature but also incorporate larger and more diverse samples. The inclusion of a variety of age groups and ethnic backgrounds would be particularly beneficial to further elucidate the intricate relationships between metabolic syndrome, hyperuricemia, and broader health outcomes.

## 5. Conclusions

The prevalence of MetS in our study population was high. High serum uric acid levels are independently associated with the presence of MetS among the middle-aged and elderly Taiwanese population. Due to the potential limitations of this study, further prospective cohort studies are needed to confirm our findings.

## Figures and Tables

**Table 1:** General characteristics of the study population according to tertiles of uric acid levels.

Uric Acid Levels													
	Total			Low			Middle			High			
Variables	(n = 400)			(n = 130)		(<5.1)	(n = 138)		(5.1–6.2)	(n = 132)		(>6.2)	*p* Value
Age (year)	64.47	±	8.45	64.80	±	7.90	63.26	±	8.48	65.39	±	8.84	0.10
Gender (male), n(%)	141		35.2%	29		22.3%	36		26.1%	76		57.6%	<0.001
SBP (mmHg)	129.50	±	16.71	126.43	±	17.32	130.64	±	16.36	131.33	±	16.16	0.04
DBP (mmHg)	76.93	±	11.36	74.13	±	10.20	77.49	±	11.43	79.11	±	11.89	0.001
BMI (kg/m^2^)	24.55	±	3.57	23.45	±	3.66	24.86	±	3.08	25.30	±	3.71	<0.001
Waist circumference (cm)	85.07	±	9.68	81.51	±	8.93	85.05	±	8.77	88.58	±	10.07	<0.001
ALT (U/L)	22.63	±	12.95	20.80	±	10.48	23.00	±	12.87	24.03	±	14.98	0.12
FPG (mg/dL)	96.23	±	25.73	95.18	±	22.25	95.23	±	21.13	98.32	±	32.50	0.52
HDL-C (mg/dL)	54.43	±	13.93	58.93	±	14.57	55.01	±	12.67	49.39	±	12.98	<0.001
LDL-C (mg/dL)	118.37	±	32.11	116.48	±	28.60	121.65	±	32.54	116.79	±	34.79	0.33
TC (mg/dL)	197.15	±	35.71	195.35	±	33.82	200.04	±	36.41	195.88	±	36.83	0.50
TG (mg/dL)	122.07	±	65.97	99.62	±	47.91	117.08	±	53.87	149.41	±	81.73	<0.001
Uric acid (mg/dL)	5.75	±	1.41	4.30	±	0.53	5.60	±	0.34	7.33	±	1.03	<0.001
Alcohol drinking, n (%)	75		18.8%	14		10.8%	25		18.1%	36		27.3%	0.003
Current smoking, n (%)	43		10.8%	8		6.2%	11		8.0%	24		18.2%	0.003
Regular exercise, n (%)	328		82.0%	110		84.6%	112		81.2%	106		80.3%	0.63
Metabolic syndrome, n (%)	143		35.8%	30		23.1%	52		37.7%	61		46.2%	<0.001
HTN, n (%)	201		50.3%	53		40.8%	70		50.7%	78		59.1%	0.01
DM, n (%)	79		19.8%	24		18.5%	29		21.0%	26		19.7%	0.87
Hyperlipidemia, n (%)	260		65.0%	75		57.7%	87		63.0%	98		74.2%	0.02

**Notes:** Data are expressed as the mean ± SD for continuous variables and as n (%) for categorical variables. **Abbreviations:** hypertension (HTN), diabetes mellitus (DM), body mass index (BMI), systolic blood pressure (SBP), diastolic blood pressure (DBP), total cholesterol (TC), triglyceride (TG), fasting plasma glucose (FPG), low-density lipoprotein (LDL-C), high-density lipoprotein (HDL-C), and alanine transaminase (ALT).

**Table 2:** Pearson’s coefficient between uric acid and cardiometabolic variables.

	Uric Acid	
Variables	Pearson’s Coefficient	*p* Value
Age (year)	0.02	0.67
SBP (mmHg)	0.13	0.01
DBP (mmHg)	0.23	<0.001
BMI (kg/m^2^)	0.21	<0.001
Waist circumference (cm)	0.30	<0.001
ALT (U/L)	0.10	0.04
FPG (mg/dL)	0.05	0.31
HDL-C (mg/dL)	−0.30	<0.001
LDL-C (mg/dL)	0.02	0.69
TC (mg/dL)	0.02	0.67
TG (mg/dL)	0.33	<0.001

**Abbreviations:** body mass index (BMI), systolic blood pressure (SBP), diastolic blood pressure (DBP), triglyceride (TG), total cholesterol (TC), fasting plasma glucose (FPG), low-density lipoprotein (LDL-C), high-density lipoprotein (HDL-C), and alanine transaminase (ALT).

**Table 3:** Multivariate logistic regression models on uric acid levels related to the MetS risk among adjusted populations.

Variables		Unadjusted		Adjusted		
	OR	(95% CI)	*p* Value	OR	(95% CI)	*p* Value
Low	1.00	-	-	1.00	-	-
Middle	2.02	(1.18–3.44)	0.01	1.73	(0.95–3.16)	0.07
High	2.86	(1.68–4.88)	<0.001	2.48	(1.31–4.71)	0.01
Adjusted for age, sex, alcohol drinking, smoking, BMI, regular exercise, ALT, and TC						

**Abbreviations:** odds ratio (OR), confidence interval (CI), body mass index (BMI), total cholesterol (TC), and alanine transaminase (ALT).

**Table 4:** Area under the receiver operating characteristic (ROC) curve (AUC), sensitivity, and specificity using the optimized cutoff point for uric acid in predicting MetS.

Variables	AUC (95% CI)	*p* Value	Cut-Off Point	Sensitivity	Specificity
Uric acid	0.62 (0.56–0.68)	<0.001	5.62	0.61	0.59

**Abbreviations:** ROC (receiver operating characteristic); CI (confidence interval).

## Data Availability

The authors declare that the data supporting the findings of this study are available within the paper.
